# Molecular oxygen as a probe molecule in EPR spin-labeling studies of membrane structure and dynamics

**DOI:** 10.3390/oxygen2030021

**Published:** 2022-08-04

**Authors:** Witold K. Subczynski, Justyna Widomska, Marija Raguz, Marta Pasenkiewicz-Gierula

**Affiliations:** 1 Department of Biophysics, Medical College on Wisconsin, Milwaukee, United States; 2 Department of Biophysics, Medical University of Lublin, Lublin, Poland; 3 Department of Medical Physics and Biophysics, University of Split School of Medicine, Split, Croatia; 4 Department of Computational Biophysics and Bioinformatics, Jagiellonian University, Krakow, Poland

**Keywords:** Molecular oxygen, lipid spin labels, EPR, lipid bilayer membranes, membrane fluidity, membrane domains, cholesterol

## Abstract

Molecular oxygen (O_2_) is the perfect probe molecule for membrane studies carried out using the saturation recovery EPR technique. O_2_ is a small, paramagnetic, hydrophobic enough molecule that easily partitions into a membrane’s different phases and domains. In membrane studies, the saturation recovery EPR method requires two paramagnetic probes: a lipid-analog nitroxide spin label and an oxygen molecule. The experimentally derived parameters of this method are the spin-lattice relaxation times (*T*_1s_) of spin labels and rates of bimolecular collisions between O_2_ and the nitroxide fragment. Thanks to the long *T*_1_ of lipid spin labels (from 1 to 10 μs), the approach is very sensitive to changes of the local (around the nitroxide fragment) O_2_ diffusion-concentration product. Small variations in the lipid packing affect O_2_ solubility and O_2_ diffusion, which can be detected by the shortening of *T*_1_ of spin labels. Using O_2_ as a probe molecule and a different lipid spin label inserted into specific phases of the membrane and membrane domains allows data about the lateral arrangement of lipid membranes to be obtained. Moreover, using a lipid spin label with the nitroxide fragment attached to its head group or a hydrocarbon chain at different positions also enables data about molecular dynamics and structure at different membrane depths to be obtained. Thus, the method can be used to investigate not only the lateral organization of the membrane (i.e., the presence of membrane domains and phases), but also the depth-dependent membrane structure and dynamics, and, hence, the membrane properties in three dimensions.

## 1. Introduction

The lipid bilayer constitutes a basic structural element and the hydrophobic barrier of each biological membrane. The first biological membrane model [1, 2] predicted that it consisted of a rigid lipid bilayer to which membrane proteins were attached. In the more recent fluid-mosaic model, the lipid bilayer was a two-dimensional fluid medium in which membrane proteins were freely floating [3]. The current models present a membrane as a multi-domain supramolecular structure. The lipid membrane domains are transient and can form different thermotropic and lyotropic phases. Nevertheless, each domain is fluid. Fluidity is understood as motional freedom of lipid molecules and *trans-gauche* isomerization along their hydrocarbon chains. Lipid bilayer fluidity was pointed out by Robertson [4] as the crucial property of the membrane indispensable for fulfilling its biological functions. The lipid bilayer of the animal cell membrane is composed of a variety of phospholipids (PL) and cholesterol (Chol) and accommodates different integral and peripheral membrane proteins. This compositional complexity requires lateral organization of the membrane. It is not surprising that the cellular lipid membrane shows the self-organizing capacity of sorting its compartments into domains with different sizes and physical properties. Additionally, in specific interactions between membrane compartments (lipid-lipid, protein-protein, and lipid-protein), the cytoskeleton network and extracellular matrix influence the lateral organization and formation of the nanoscopic heterogeneities [5, 6]. Lipid rafts [7–9] are the example of these heterogeneities that have been investigated most often. Pike [10] defined lipid rafts as “small (10–200 nm), heterogeneous, highly dynamic, sterol- and sphingolipid-enriched domains that compartmentalize cellular processes.” However, nanodomains that are smaller than 10 nm exist within the surrounding lipid environment [11, 12]. Thus, the current opinion of membranes is that they are a highly dynamic structure formed by heterogeneous domains with different diameters, properties, and functions.

The membrane organization, realized on different length scales and varied with time, has been investigated by diverse methods [13–20]. One of them is the dual-probe saturation recovery (SR) electron paramagnetic resonance (EPR) spin-labeling method, where molecular oxygen (O_2_) is a probe molecule [21–25]. This method allows discrimination of membrane domains and phases, and quantitatively characterizes them not only laterally, but also as a function of the membrane depth. These quantitative descriptions of the three-dimensional membrane structure and properties are important to understand different membrane functions better. This review describes the lateral organization of lipid bilayers as well as the lipid bilayer portion of intact biological membranes studied using mainly the EPR spin-labeling methods and O_2_ as a probe molecule.

## 2. Molecular Oxygen

The small size (MW = 32) and proper hydrophobicity of O_2_ allow it to diffuse into different supramolecular structures, including membrane phases and domains. O_2_, with its triplet ground state (S = 1), is paramagnetic. Triplet oxygen is a relatively weak oxidant and a stable paramagnetic species. The EPR signal of gaseous O_2_ is strong [25–27]; however, no EPR spectra can be recorded for O_2_ dissolved in liquids at physiological temperatures (see more explanation in [24]). Fortunately, in a spin labeled, hydrated, oxygenated sample, collisions between dissolved O_2_ (invisible through EPR) and the spin-label (visible through EPR) take place and change the spectral characteristics of the spin label. To monitor the changes in EPR spectra, either the *T*_1_-sensitive or *T*_2_-sensitive approach can be used. Both of them provide quantitative information [24]. Here, the main focus is on the *T*_1_-sensitive method.

## 3. *T*_1_-Sensitive Method for Monitoring the Oxygen Diffusion-Concentration Product

*T*_1_ is the time constant of spin-lattice relaxation, and *T*_2_ is the time constant of spin-spin relaxation. The SR EPR method is a pulse technique used to obtain the spin lattice relaxation time of a paramagnetic species. In SR EPR studies of lipid bilayers, the measured relaxation time is the *T*_1_ of a nitroxide spin label. Because the *T*_1_ is 10 to 1,000 times longer (usually 1–10 μs) than the *T*_2_, the *T*_1_-sensitive approach enables investigation of processes that occur on a longer time scale. Another advantage of the *T*_1_-sensitive method is that it can be used to measure any spin-labeled (like proteins) or spin-probed (like membranes) samples. Collisions of O_2_ with the nitroxide moieties induce changes in *T*_1_, and for this reason O_2_ is used as a probe molecule. Because the *T*_1_ of O_2_ is much shorter than that of a nitroxide spin label, each O_2_-nitroxide collision induces instantaneous relaxation of the nitroxide. In the *T*_1_-sensitive method, lipid-analog spin labels with the nitroxide moiety attached to a particular position along the acyl chain are introduced into sub-membrane structures such as phases and domains. Collision-induced changes in *T*_1_ allow the diffusion-concentration product for O_2_ to be obtained at different membrane microenvironments (horizontal) [22, 28] and at different membrane depths (vertical) [16, 29]. This provides information about the spatial (three-dimensional) organization of the membrane and dynamics of lipids around the nitroxide group.

Alternative approaches to measuring the oxygen-diffusion concentration products include fluorescence quenching [30, 31], EPR spin-label line broadening [32–34], and nuclear magnetic resonance proton spin-lattice relaxation [35–37]. All of these techniques measure the frequency of collision rates between the probe molecule and molecular oxygen, and all have been applied to study oxygen in phospholipid bilayers. The advantages of the *T*_1_-sensitive approach over *T*_2_-sensitive approach are well documented in the literature [38–40] and are discussed at the beginning of this section. One difficulty with fluorescence quenching is that the theory of quenching by oxygen is very imperfectly developed. The process appears to be a “strong encounter” type—the probability of quenching is unity for each collision. The nuclear magnetic resonance method is a “weak encounter” type—the probability of an observable event per collision is much less than unity. Quantitative measurements are difficult, and this method also may be too slow if one wishes to observe the time evolution of the O_2_ diffusion-concentration product.

## 4. Methods of Controlling Oxygen Concentration (Oxygen Partial Pressure) in Investigated Samples

**Figure 1.**
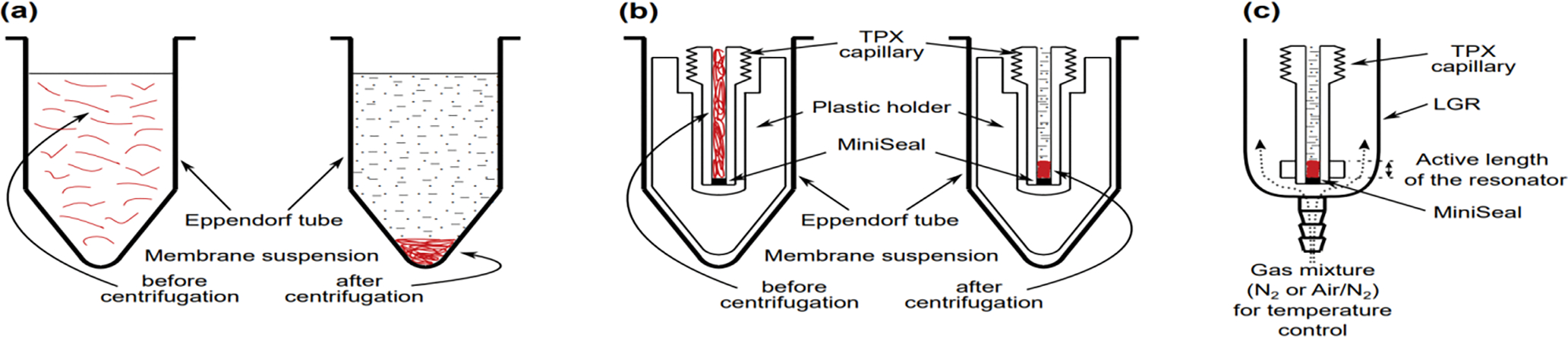
Schematic drawing showing the handling of samples with a small amount of a biological material for EPR measurements. (a) The first step: concentrating of the diluted sample by centrifugation in Eppendorf tubes to the volume of a TPX capillary. (b) The second step: further of concentrating the sample to match the sample length in the TPX capillary with the active length of the resonator. (c) The third step: positioning the TPX capillary inside the LGR with the sample located exactly in the active volume of the resonator. In the resonator, the sample can be equilibrated with the appropriate air/nitrogen mixture.

Application of SR EPR in membrane studies that involve O_2_ as a probe molecule requires precise control of oxygen concentration or, more accurately, oxygen partial pressure in the membrane at the temperature at which the experiment is performed. For this purpose, the sample tubes (which are capillaries machined from a methylpentene polymer known as TPX) were used [41, 42]. The walls of this capillary are permeable to gases (oxygen, nitrogen, and other gases) but in practice are impermeable to water. The sample is placed in such a capillary and positioned inside the loop-gap resonator (LGR); then, it is equilibrated with an appropriate air/nitrogen mixture adjusted using flowmeters, which also are applied to control temperature (Fig. 1c). For EPR measurements at Q-band (35 GHz) and W-band (94 GHz), a thin wall Teflon capillary is used. Because this capillary is flexible, a special holding must be applied to properly position it inside the LGR (see [42] for more explanation).

The LGR [43] improves the sensitivity of the EPR method in the case of a small sample. LGRs constructed for the X-band EPR spectrometer (9.4 GHz) have an active sample volume of a few microliters and an active length of 2.5–5 mm [43–45]. LGRs constructed for the Q-band and W-band spectrometers have a sample volume of 30 nL and an active length of 1 mm [42, 44, 45]. The handling samples of such small volumes is described in [42]. In brief, the sample is concentrated by centrifugation in an Eppendorf test tube (Fig. 1a), transferred to a capillary, and concentrated by centrifugation to match the length of the pellet at the bottom of the capillary with the active length of the LGR (see scheme in Fig. 1b).

As is shown in Fig. 1c, samples such as spin-labeled liposomes or biological membranes can be thoroughly equilibrated with the needed partial pressure of O_2_ at a chosen temperature. The partial pressure of O_2_ in the equilibrated sample is the same at all points across the sample. However, the local O_2_ concentration can differ significantly (see profiles of oxygen transport parameter presented below). The local O_2_ concentration is determined by the local O_2_ solubility coefficient multiplied by the O_2_ partial pressure with which the sample is equilibrated. However, it cannot be measured directly. Thus, observed changes of the EPR spectral parameters of spin labels induced by collisions with O_2_ (here, they are changes in the *T*_1_ of spin labels) depend on the local O_2_ concentration (multiplied by the local O_2_ diffusion coefficient) and are simply proportional to the oxygen partial pressure.

The home-built X-band SR EPR spectrometers used for *T*_1_ measurements received a few major hardware improvements in order to acquire the data presented in this paper. The pump arm in the SR system is now capable of delivering a pulse width as narrow as 10 ns at a 1 W power level to the LGR. The availability of this level of pump power ensures saturation of the sample with the narrow pump pulse widths needed to detect the faster components present in multiexponential signals. In most cases described in this article, the spin-lattice relaxation times, *T*_1_s, of spin labels were determined by analyzing the SR signals of the most intensive central line obtained by short-pulse (300 ns) experiments. As indicated in Ref. [46], for short-pulse SR experiments (which is the case described here), spectral diffusion processes, such as molecular tumbling and nuclear spin relaxation, frequently contribute to the recovery curve. Thus, in principle, the measured spin-lattice relaxation times are the effective spin lattice relaxation times. The field jumps can occur in less than 200 μs at the full 34 G amplitude. The field amplitude is sufficient to jump completely off the low field line of the nitroxide in a downfield direction to ensure instrumental artifacts are fully subtracted from magnetic resonance. The receiver dead time after the pump pulse was reduced to 100 ns from the previous value of 300 ns. This is the receiver delay or blanking time required to protect the receiver from the effects of the saturating pump pulse. This reduction greatly aids data analysis of signals with double-exponential content, particularly when fitting signals contain faster components (see Ref. [47] for more details).

## 5. The Oxygen Transport Parameter (Outline of Theory)

To evaluate the rate of collisions between spin labels and O_2_, Kusumi et al. introduced a convenient parameter named the oxygen transport parameter (OTP) [48]. Even though the name of the parameter contains the word “transport,” the process it describes is not related to active transport across or within the membrane.

The OTP is defined as follows:

(1) $$\mathrm{OTP}(x)=T_{1^{-1}}(\text{Air},\;x)-T_{1^{-1}}\left(\;{\text{N}}_{2},\;x\right)$$

Here, $T_{1^{-1}}\text{s}$ are the spin-lattice relaxation rates of the nitroxide moiety of the spin label positioned at the depth *x* (distance from the membrane center) of the membrane equilibrated with air (Air) and nitrogen (N_2_). Thus, to get the value of the OTP, two SR EPR signals have to be measured, one of a deoxygenated sample and the other of that equilibrated with air. *T*_1_ values are obtained from fitting each of these signals to a single exponential function [48]. The value of the OTP is normalized to the atmospheric partial pressure of oxygen in air surrounding the sample capillary, namely 159.6 mmHg. It happens quite often that in the presence of air, the relaxation process is too fast to allow the recovery signal to be recorded. Thus, to increase the accuracy of $T_{1}^{-1}(\text{Air},\;\text{x})$ measurements, its value is obtained by extrapolating the linear plot of $T_{1^{-1}}(f\text{Air},\;\text{x})$ as a function of the fraction *f* of air in the equilibrating gas mixture to pure air (*f* = 1) [22, 49]. As illustrated in Eq. 1, the contributions of all relaxations, except the Heisenberg exchange that occurs during collisions between the spin labels and O_2_, are canceled. The only possible contribution is the effect of molecular oxygen on the nuclear spin relaxation. However, as was indicated at the end of the Sect. 3, this interaction is of a “weak encounter” type—the probability of an observable event per collision is much less than unity, so it can be excluded.

The OTP can also be expressed by the Smoluchowski equation, which in the case of a spherical particle, takes the following form:

(2) $$\mathrm{OTP}(x)=A\left(D_{\text{SL}}(x)+D(x)\right)C(x),\;A=4\pi pr\circ$$

Here, *D*(*x*) and *C*(*x*) are, respectively, the O_2_ diffusion coefficient and the O_2_ concentration at the depth *x* (around the nitroxide moiety) in the membrane equilibrated with air; *D*_SL_(*x*) is the diffusion coefficient of the lipid spin label; *r*_o_ the interaction distance between O_2_ and the nitroxide moiety of the spin label, equal to 4.5 Å [50]; and *p* is the probability that a spectroscopically observable event occurs when a collision takes place (see [21, 48] for more explanation). In the SR EPR methodology, *p* is assumed to be 1.

Since the *D*(*x*) (diffusion coefficient of O_2_) in membranes is much greater than the *D*_SL_(*x*) (diffusion coefficient of the lipid spin label), the latter can be omitted in Eq. 2, which now takes a simpler form:

(3) OTP(x)=AD(x)C(x)

The combination of Eq. 1 and Eq. 3 provides the method of obtaining the O_2_ diffusion-concentration product, *D*(*x*)*C*(*x*), from the SR EPR measurements. It should be stressed here that when using experimental methods, it is not possible to factor this product into *D*(*x*) and *C*(*x*). It was shown that *A* is independent of the kind of a spin label, local hydrophobicity, and local viscosity of the environment around the nitroxide moiety [24, 51, 52]. The profiles of the OTP (and thus the O_2_ diffusion-concentration product) are shown in Fig. 4b, Fig. 5c, Fig. 7a and Fig. 8. The following statement, made by Kusumi et al. [48], is significant to this review: “The OTP is a useful monitor of membrane fluidity that reports on translational diffusion of small molecules.”

**Figure 2.**
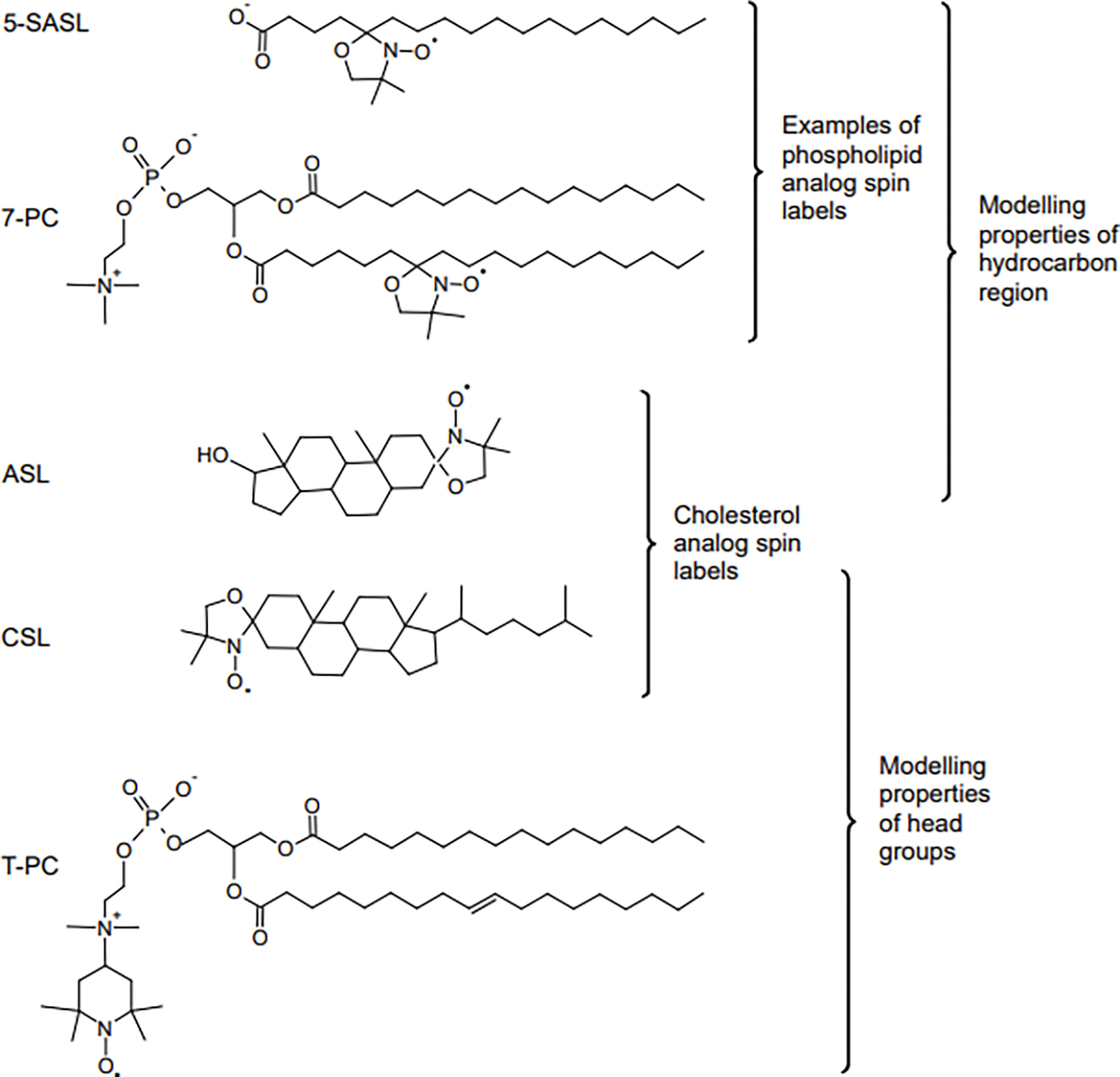
Chemical structures of selected lipid spin labels used for membrane studies. The phospholipid analogs 7-doxylstearic acid spin label (5-SASL) and 1-palmitoyl-2-(7-doxylstearoyl)phosphatidylcholine (7-PC) models properties of the membrane hydrocarbon region, cholesterol analogues cholestane spin label (CSL) and androstane spin label (ASL) model the behavior of Chol molecules in the lipid bilayer, and the phospholipid analog tempocholine-1-palmitoyl-2-oleoylphosphatidic acid ester (T-PC) models properties of the head groups region.

Ashikawa et al. [28] developed a discrimination by oxygen transport (DOT) method that allows domains and phases in biological and model membranes to be discriminated with the use of OTP. In this approach, lipid-analog spin labels have to be introduced into these domains and phases. In most cases, the lipid spin labels themselves cannot discriminate these membrane substructures because their *T*_1_s have similar values in both environments; however, the addition of O_2_ to the sample differentiates their *T*_1_s. The chemical structures of selected spin labels, as well as their approximate localizations within the lipid bilayer, are presented in Fig. 2.

**Figure 3.**
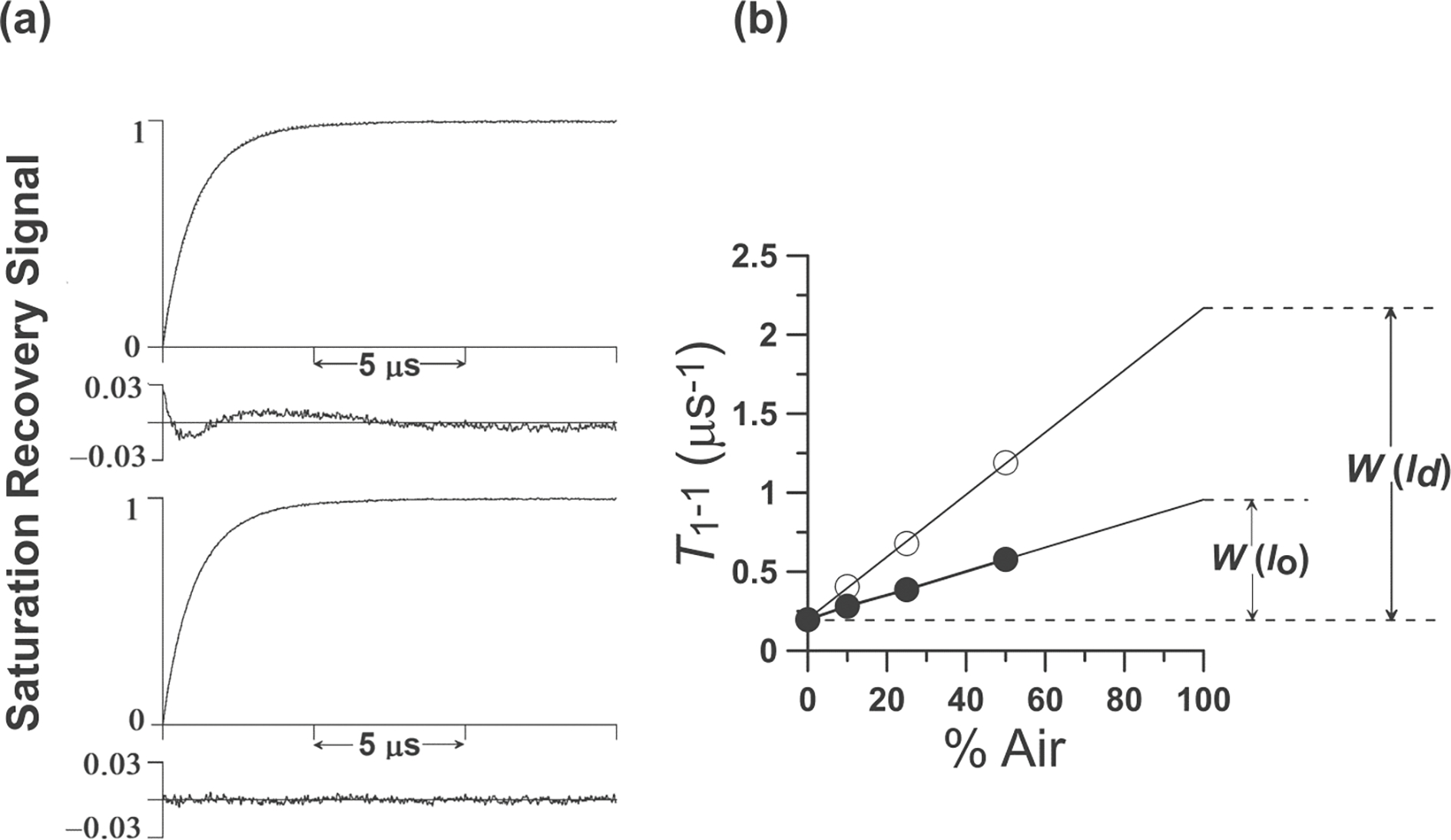
**(a)** Representative SR signal from 5-PC in the DMPC bilayer containing 20 mol% Chol obtained at 30°C for the sample equilibrated with 50% air. In the deoxygenated sample, a single exponential signal is observed with a time constant of 5.10 μs (data not shown). In the presence of oxygen, fitting the search to a single exponential mode is unsatisfactory as shown by the residual (upper panel). The fit, using the double-exponential mode (time constants of 1.73 and 0.84 μs), is excellent (lower panel). The double-exponential fit is consistent with two immiscible domains (phases) with different OTPs that are present at these conditions. (We assigned them to the *l*_d_ phase and *l*_o_ phases.) **(b)** Plot of $T_{1^{-1}}$ for 5-PC in the *l*_o_ and *l*_d_ phases in a DMPC membrane containing 20 mol% Chol as a function of air fraction in the equilibrating gas mixture. Experimental points show a linear dependence up to 50% air, and extrapolation to 100% air is performed as a way of calculating OTPs in the *l*_o_ and *l*_d_ phases.

In the case of a two-phase membrane or a membrane with one kind of domains that coexist with the bulk phase, the recorded SR EPR signal is the sum of two single-exponential functions (double exponential curve), each representing one kind of spin label environment (see Fig. 3). Because the *T*_1_s of lipid spin labels measured in the absence and presence of O_2_ range between 0.1 and 10 μs, the exchange rates of the spin labels between the coexisting domains must be slower than or comparable to these times in order to discriminate between them. If the exchange rate is fast (faster than 10^7^ s^−1^), discrimination is not possible because the measurement provides a *T*_1_ that is the average of all the *T*_1_s. For exchange rates slower than 10^4^ s^−1^, the domains can be treated as completely separate from the bulk domains, and the OTPs are their true parameters. For exchange rates between 10^4^ and 10^7^ s^−1^, the spin label exchange rates, in principle, can be evaluated using the theory developed by Kawasaki et al. [53].

For noninteracting, separated domains, the double exponential SR EPR signals with spin lattice relaxation rates $T_{1^{-1}}(\text{Air},\;\text{FOT})$ and $T_{1^{-1}}(\text{Air},\;\text{SLOT})$ are observed in the domain with the fast OTP (FOT domain) and that with the slow OTP (SLOT domain). Thus, the OTP in each domain is as follows:

(4) $$\text{OTP}(\text{FOT})=T_{1^{-1}}(\text{Air},\;\text{FOT})-T_{1^{-1}}\left(\;{\text{N}}_{2},\;\text{FOT}\right)$$

(5) $$\text{OTP}(\text{SLOT})=T_{1^{-1}}(\text{Air},\;\text{SLOT})-T_{1^{-1}}\left({\text{N}}_{2},\;\text{SLOT}\right)$$

**Figure 4.**
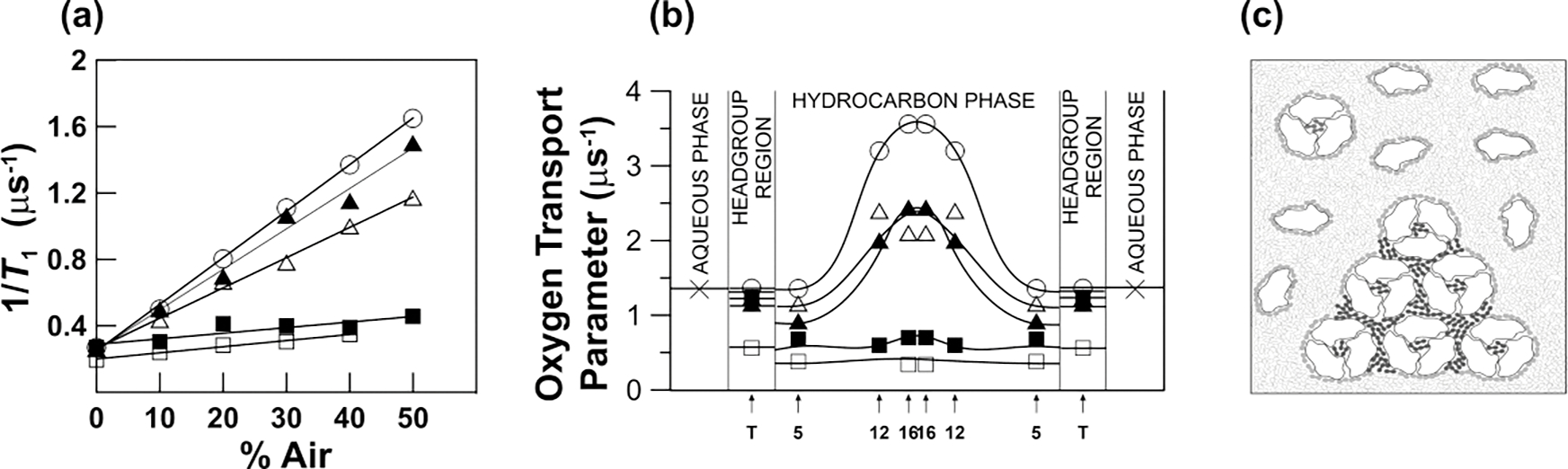
Steps used in the discrimination of membrane domains using the DOT method. **(a)** Spin lattice relaxation rates of 1-palmitoyl-2-(12-doxylstearoyl)phosphatidylcholine (12-PC) plotted as a percentile of air in the gas mixture equilibrating the membrane suspension at 30°C. Symbols are for the DMPC bilayer without BR (○), with BR/DMPC = 1/80 (Δ), with BR/DMPC = 1/40 (▲,■), and for purple membranes isolated from *Halobacterium halobium* (□). $T_{1^{-1}}$ values were extrapolated to 100% air and OTP was calculated for each domain. As indicated in Eq. 1, the SR signals obtained for the DMPC bilayer without BR, with BR/DMPC = 1/80, and for purple membranes were successfully fitted to single exponentials, giving single values of the OTP for all spin labels (using Eq. 1). SR signals obtained for the DMPC bilayer with BR/DMPC = 1/40 were successfully fitted only to double exponential functions, giving two values of the OTP for each spin label (using Eqs. 4 and 5). **(b)** Profiles of the OTP values obtained at 30°C from different PL spin labels across the DMPC bilayer without BR (○), with BR/DMPC = 1/80 (Δ), with BR/DMPC = 1/40 (▲,■), and across purple membranes (□). When BR is in the monomeric form, only one bulk-plus-boundary lipid domain is present (Δ). When BR is aggregated, two lipid domains coexist: bulk-plus-boundary domain (▲) and trapped lipid domain (■). Arrows indicate approximate locations of nitroxide moieties of n-PCs and n-SASLs used in these investigations. T indicates T-PC. The symbol × indicates OTP in the aqueous phase. **(c)** Schematic drawing of the lateral organization of bacteriorhodopsin and lipid molecules in the reconstituted membrane of BR and DMPC at a BR/lipid ratio of 1/40. Phospholipid molecules are indicated as an open and closed figure-eight-shaped phospholipid cross section. Phospholipids in the bulk domain are open, in the boundary are grey, and in the SLOT domain (trapped-lipid domain) are dark. Lipids in the SLOT domain are trapped between trimers and oligomers of trimers of the BR. The schematic shapes of molecules are drawn on the base of the electron microscopy studies [54]. Data for Fig. 4a and Fig. 4b are reproduced with permission from Ref. [55]. Copyright 2022, American Chemical Society.

As indicated above, most often $T_{1^{-1}}\left(\;{\text{N}}_{2},\;\text{FOT}\right)=T_{1^{-1}}\left(\;{\text{N}}_{2},\;\text{SLOT}\right)$ and the spin label alone cannot discriminate coexisting domains (see Fig. 4a). In the described case, namely reconstituted membranes of bacteriorhodopsin (BR) and dimyristoylphosphatidylcholine (DMPC), the same lipid spin label is used to discriminate domains and give the OTP at certain domain depths. Using lipid spin labels with nitroxide fragments positioned at different depths in the membrane, transmembrane profiles of the OTP can be received in coexisting domains without the need for their physical separation (Fig. 4b). The schematic illustration of the coexisting domains in reconstituted membranes of BR and DMPC is shown in Fig. 4c.

## 6. Molecular Oxygen Differently Monitors Membrane Fluidity and Dynamics of Acyl Chains

An order parameter is a normalized parameter that indicates the degree of order of a system. An order parameter of 0 indicates disorder; the absolute value in the ordered state is 1 [56]. The order of the acyl chains across a fluid-phase phospholipid bilayer can be obtained directly from EPR spectra of lipid-analog spin labels. For this reason, the nitroxide moiety of the spin label is attached to the subsequent carbon atoms along the hydrocarbon chain of the label, and the EPR spectrum for each position of the nitroxide is recorded. From each spectrum, the value of the order parameter is derived. The profile of the order parameter across the bilayer is often called the fluidity profile. However, as the order parameter measures the angular amplitude of the wobbling motion of the chain fragment to which the nitroxide moiety is rigidly attached, it is a static parameter [57]. Thus, the profile of the order parameter informs only indirectly about the motional freedom of the nitroxide moiety at a certain bilayer depth.

Dynamic parameters, which explicitly characterize time-dependent processes, e.g., diffusion, describe the membrane fluidity much better than the order parameter. Information about time scales of axial rotation (about the long axis) and wobbling (about the perpendicular axis) of lipid molecules or their fragments in the bilayer can be obtained from respective rotational diffusion coefficients of lipid-analog spin labels. The rotational diffusion coefficient can be derived from the spin label EPR spectrum by means of the microscopic order and macroscopic disorder (MOMD) model [58–60]. The profile of the spin label rotational diffusion coefficient can be obtained in the analogy to the order parameter profile and is an actual quantitative measure of the membrane fluidity [61, 62]. The rotation of the certain fragment of the acyl chain, to which the monitoring nitroxide group is rigidly attached, is the result of a cumulative effect of all rotations that take place simultaneously at different positions along the chain. SR EPR measurements on deoxygenated spin labeled membranes provide values of spin-lattice relaxation rates $\left(T_{1^{-1}}\;\text{s}\right)$, which depend primarily on the rate of rotational motion of the nitroxide moiety [63–65]. It was shown that $T_{1^{-1}}\;$ can also be used as a convenient parameter that monitors the membrane dynamics at different depths and provides the profile of membrane fluidity [61, 62]. As indicated in this review, membrane fluidity can also be estimated by measuring the diffusion within the membranes of small probe molecules, such as O_2_. This approach differs significantly from measurements of fluidity of membrane acyl chains. It is not affected by the cumulative effect mentioned above and allows more detailed information about membrane fluidity to be obtained with a much greater spatial resolution.

O_2_ dissolved in the membrane locates primary in the nonpolar bilayer core [66]. The lateral diffusion of O_2_ there was first described by Träuble (1971) [67] as its movement with migrating vacant pockets (kink conformations) and by Pace and Chan (1982) [68] as its hopping between neighboring kinks. The model of the O_2_ lateral diffusion was later extended by Subczynski et al. [69], in O_2_, to also include hopping between the vacant pockets that are formed in the bilayer as a result of the structural nonconformability of neighboring lipids. The extended model of Subczynski et al. [69] covers a wide range of packing defects that can be involved in O_2_ diffusion in the membrane. Because O_2_ diffusion is very sensitive to the dynamics of the lipid acyl chain, O_2_ as a probe molecule can provide information about the three-dimensional dynamical structure of the lipid bilayer at the sub-molecular level. On the basis of this information, bulk, boundary, and trapped lipid domains [28, 53, 70, 71]; liquid-ordered (*l*_o_) phase domains [16, 29]; and pure Chol bilayer domains (CBDs) [16, 72–77] in model and biological membranes can be discriminated.

## 7. Domain Structure of Model and Biological Membranes

### 7.1. Cholesterol Induced Phases in Lipid Bilayers

**Figure 5.**
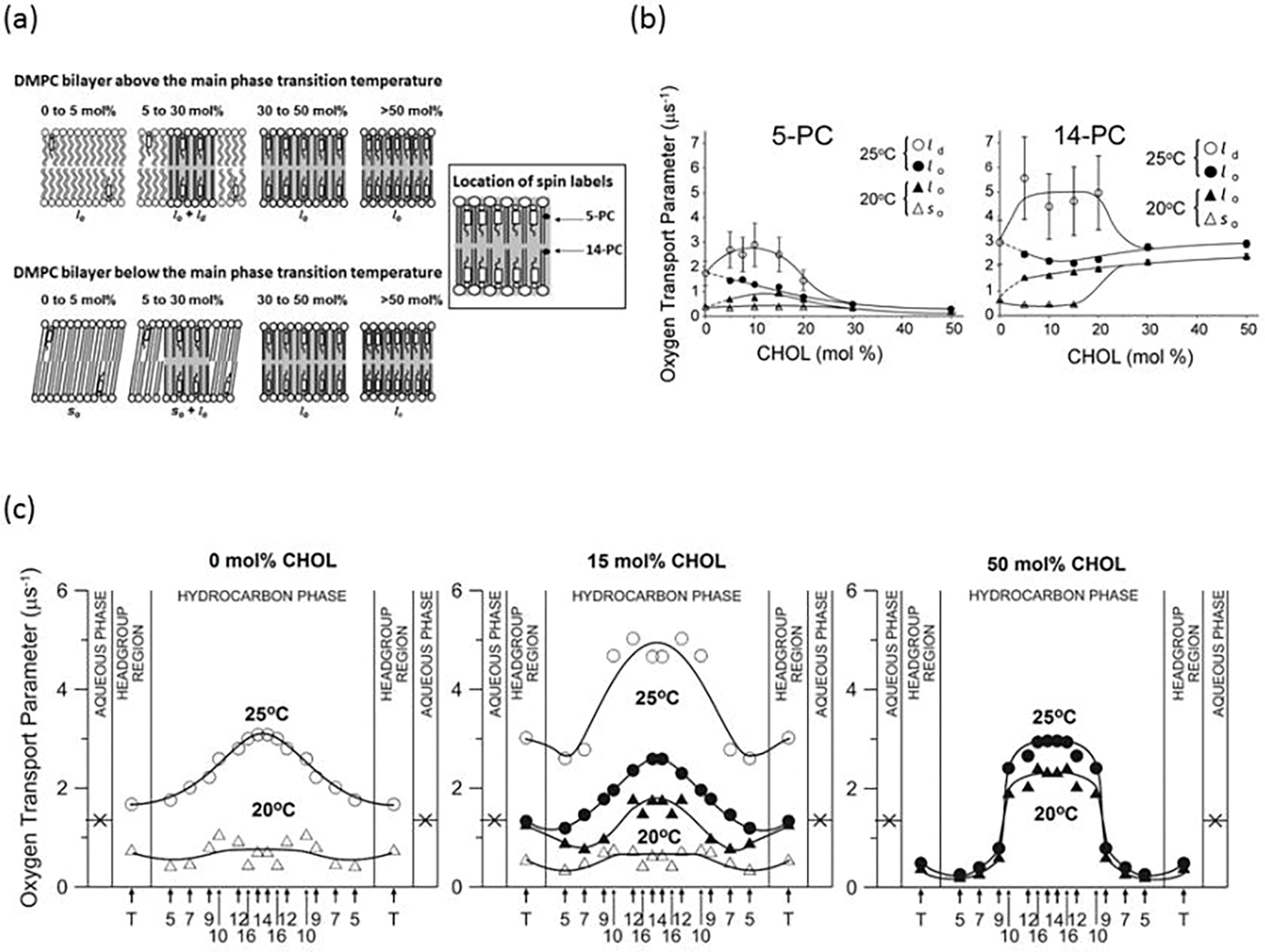
Steps used in the discrimination of membrane phases using O_2_ as a probe molecule. **(a)** Schematic drawings of membrane phases formed above (~25°C) and below (~20°C) the main phase transition temperature of the pure DMPC bilayer at different Chol contents in the Chol/DMPC mixture. Three basic bilayer phases are recognized: the solid-ordered (*s*_o_) phase, the liquid-disordered (*l*_d_) phase, and the liquid-ordered (*l*_o_) phase (indicated by in grey). At 20°C, the *s*_o_ and the *l*_o_ phases coexist with a Chol/DMPC mixing ratio between ~5 and ~30 mol% Chol. At 25°C, the *l*_d_ and the *l*_o_ phases coexist with a Chol/DMPC mixing ratio between ~5 and ~30 mol% Chol. **(b)** Plots of the OTPs obtained with 5-PC and 14-PC as a function of the Chol mixing ratio in Chol/DMPC membranes allowed indicate Chol contents at which a single *s*_o_ (between 0 and ~5 mol%), a single *l*_d_ (between 0 and ~5 mol%), and a single *l*_o_ phase exists (between ~30 and ~50 mol%) and Chol contents at which *s*_o_ and *l*_o_ phases as well as *l*_d_ and *l*_o_ phases coexist (between ~5 and ~30 mol%). Data are for 20°C and 25°C. Symbols are explained in the figures. **(c)** Profiles of OTP obtained at 20°C and 25°C across DMPC membranes without Chol, containing 15 mol% Chol, and containing 50 mol% Chol. Symbols used are (Δ) for the *s*_o_ phase, (○) for the *l*_d_ phase, and (●,▲) for the *l*_o_ phase. Arrows indicate approximate locations of nitroxide moieties of spin labels. T indicates T-PC. The symbol × indicates OTP in the aqueous phase. It does not change significantly because the temperature dependences of O_2_ diffusion and concentration in water are opposite. As shown, these profiles were obtained in single and coexisting domains and characterize their physical properties without physical separation of domains. Fig. 5b and Fig. 5c are reproduced from Ref. [29]. Copyright 2022, with permission from Elsevier.

The potential of the DOT method to identify membrane phases with PC-analog spin labels was demonstrated for Chol-induced membrane phases in the DMPC bilayer [29]. In the Chol/DMPC bilayer, three phases can be distinguished, the liquid-disordered (*l*_d_), *l*_o_, and solid-ordered (*s*_o_). The phase can exist as single or coexisting phases [29], as is schematically illustrated in Fig. 5a. In Fig. 5b, the values of the OTP measured above (25°C) and below (20°C) the main phase transition temperature (*T*_m_) of the pure DMPC bilayer (23.6°C) are presented as a function of the Chol bilayer content. Values of the OTP for the spin label with the nitroxide moiety located both close to the surface (5-PC) and close to the center (14-PC) of the Chol/DMPC bilayer (Fig. 5b) indicate that the bilayer phase remains *s*_o_ at 20°C and *l*_d_ at 25°C for Chol concentrations from 0 to ~5 mol%. With increasing Chol bilayer content, the values of the OTP in the *l*_d_ phase increase; these values do not change in the *s*_o_ phase (Fig. 5b). Between ~5 and ~30 mol% of Chol content both above and below *T*_m_, two phases coexist in the bilayer; above *T*_m_, the phases are *l*_o_ and *l*_d_ and below, *s*_o_ and *l*_o_ (Fig. 5b). Following the phase diagram presented in Fig. 7 of Ref. [74], changes in Chol content in the range between ~5 and ~30 mol% above and below *T*_m_, affect only the ration of the *l*_o_ and *l*_d_ or *s*_o_ and *l*_o_ phase fractions, respectively, without changing the Chol content in the coexisting phases (for the *l*_d_ phase and *s*_o_ phase it is ~5 mol% Chol, and for the *l*_o_ phase ~30 mol% Chol). Small changes in the OTP values with increasing Chol content suggest that the size of the *l*_o_-phase domains increases and the sizes of *l*_d_- and *s*_o_-phase domains decrease. For Chol contents between ~30 and 50 mol%, the bilayer is a single *l*_o_ phase. As shown in Fig. 5b, with increasing Chol content the values of the OTP increase in the center and decrease near the surface of the bilayer.

As mentioned, O_2_ as a relaxation agent in the DOT method allows phases in the Chol/DMPC bilayers to be discriminated. Transmembrane profiles of the OTP across the discriminated phases (see Fig. 5c) enable characterization of the physical properties of these phases; this is also the case when they coexist. Previous measurements were mostly limited to membranes consisting of single phases [78–80]. In the profiles for *l*_d_ and *s*_o_ phases that contain no Chol, the values of OTP differ at any membrane depth only by a factor of 2–4, which is a somewhat surprising result. Also, the values of OTP for the *l*_d_ phase containing ~5 mol% Chol are much greater at any membrane depth than those for the *l*_d_ phase without Chol. It was shown previously [81] that the amount of Chol <5 mol% increases the acyl chain dynamics (increase *gauche-trans* isomerization) in the *l*_d_ phase, and when Chol content is greater than 5 mol%, the dynamics decreases.

The most significant and interesting part of the research presented in [29] is the characterization of the physical properties of the *l*_o_ phase under the condition when two phases *l*_o_ and *l*_d_ coexist in the bilayer. It is because, supposedly, raft domains may be formed through lipid–lipid interactions that form the *l*_o_ phase-like domains within the *l*_d_ bulk phase bilayer [82]. In Fig. 5c, profiles of the OTP across the *l*_o_ phase in the Chol/DMPC bilayers are presented for three major cases: when the *l*_o_ phase coexists with the *l*_d_ phase (*l*_o_ phase containing ~30 mol% Chol), when the *l*_o_ phase coexists with the *s*_o_ phase (*l*_o_ phase containing ~30 mol% Chol), and across a single *l*_o_ phase saturated with Chol (containing 50 mol% Chol). The OTP profile across the *l*_o_ phase containing ~30 mol% Chol is not much different from that across the membrane without Chol obtained at the same (25°C) temperature (*l*_d_ phase). The increase in Chol concentration in *l*_o_ phase up to the saturation limit (50 mol%) changes drastically the profile. The OTP values in the region close to the surface of the membrane decrease and close to the center of the membrane increase. The sharp (3–4 times) increase in the OTP at a membrane depth between positions of the C9 and C10 carbon atoms in the DMPC acyl chains changes its profile from bell shaped (Fig. 5c) to rectangular. It is interesting that the sharp change in OTP takes place at the depth in the membrane to which the rigid, plate-like Chol structure is inserted [29, 69, 83]. The results obtained with the OTP approach partly confirm previous observations that the properties of the *l*_o_ phase are between those of the *l*_d_ and *s*_o_ phases [84]. However, this is only true for Chol content when the *l*_o_ phase coexists with the *l*_d_ or *s*_o_ phases. At higher Chol concentrations (close to the Chol saturation limit), the properties of the *l*_o_ phase are like those in the *s*_o_ phase to the depth of the C9 carbon atoms of the acyl chains and like those in the *l*_d_ phase at greater depths.

### 7.2. Cholesterol Bilayer Domain

**Figure 6.**
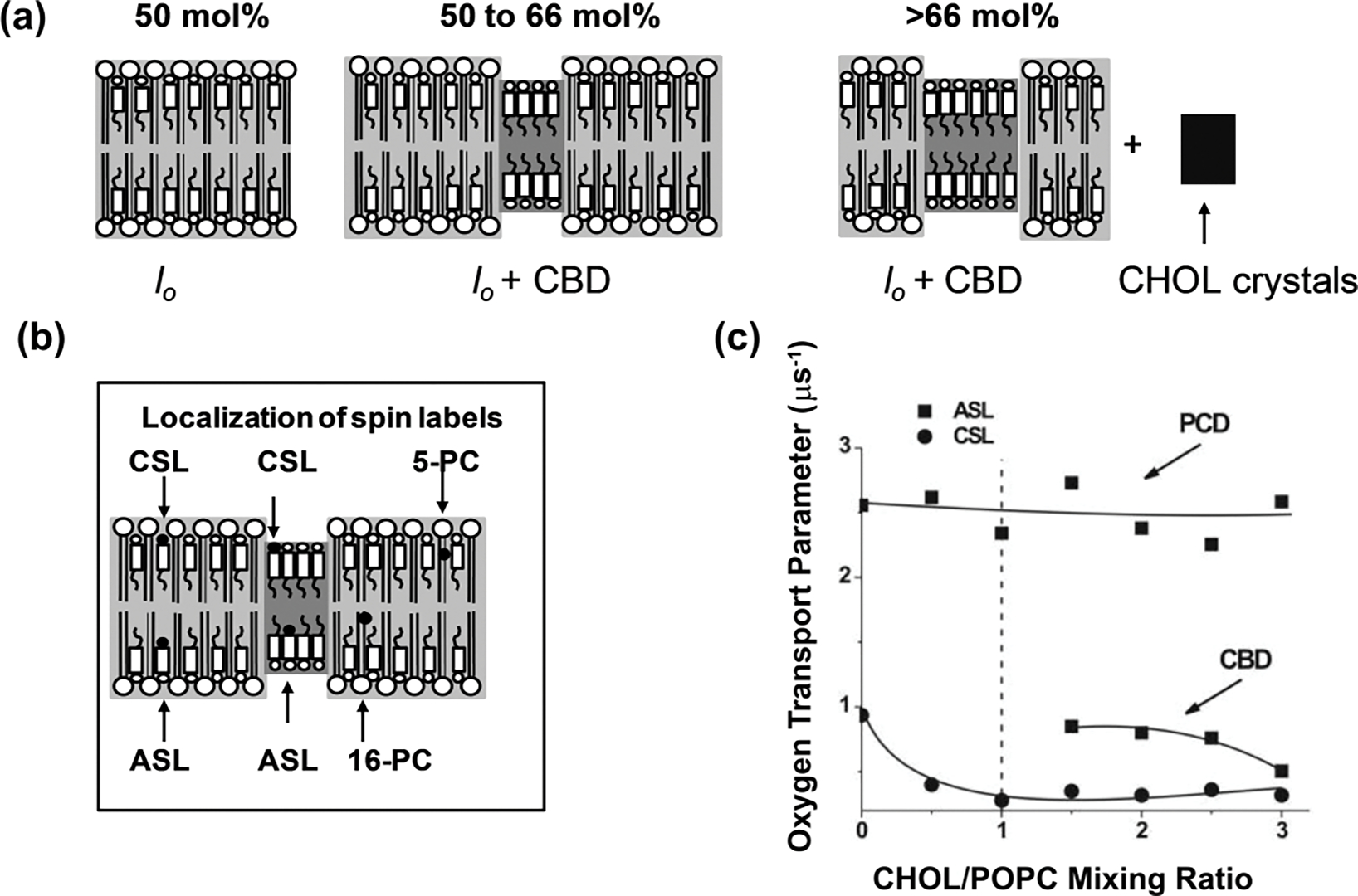
Schematic drawings and experimental data are for Chol/POPC membranes formed using the film deposition method [73]. **(a)** Schematic drawings of different membrane structures that can form at Chol contents exceeding the Chol saturation limit in the POPC bilayer (50 mol%). At the Chol saturation limit, the POPC bilayer forms the *l*o phase. When the Chol content exceeds the 50 mol% limit, pure CBDs are formed (indicated in grey) and between 50 and 66 mol% Chol (Chol solubility threshold), CBDs are supported by the POPC bilayer saturated with Chol forming one structured *l*o phase of the POPC bilayer. The phase boundary at 66 mol% Chol separates the structured *l*o phase region from the two-phase region (structured *l*o phase of POPC and Chol crystals). **(b)** Localization of representative phospholipid spin labels (5-PC and 16-PC) as well as Chol-analog spin labels (ASL and CSL) in different membrane domains are indicated. **(c)** The values of the OTP accessibility parameter obtained with ASL and CSL in POPC-Chol bilayers are displayed as a function of the Chol/POPC mixing ratio. Note that above the Chol saturation limit (at a Chol/POPC mixing ratio of 1), ASL discriminates two domains with two different OTPs assigned to the POPC bilayer saturated with Chol and to CBD. However, CSL shows only a single value of the OTP at all investigated Chol contents. Fig. 6.c is reproduced from Ref. [73]. Copyright 2022, with permission from Elsevier.

The *l*_o_ phase of the Chol/PL bilayer can accommodate up to 50 mol% of Chol. Above this concentration the excess Chol forms pure CBDs (Fig. 6a). The method discriminating these domains is described in detail in [73] for Chol/1-palmitoyl-2-oleoylphosphatidylcholine (POPC) bilayers, and a schematic description of the method is shown in Fig. 6b. The CBD, as a pure Chol domain, can be detected only with Chol-analogs androstane spin label (ASL) and cholestane spin label (CSL), which partition into both CBD and the surrounding phospholipid bilayer (see Fig. 6b). In the absence of relaxation agents, neither ASL nor CSL can discriminate the presence of CBDs because the SR EPR signal is a single exponent for every label at any Chol content in the bilayer. Only in the presence of O_2_ does the SR EPR signal of the ASL-labeled Chol/POPC bilayer containing more than 50 mol% Chol have two clear components and provide two OTP values; one is assigned to CBDs and the other to the Chol/POPC bilayer surrounding the CBDs (see Fig. 6c). CSL does not discriminate the CBD from the bulk membrane in the presence of O_2_ because the OTP values in both coexisting domains are too close to each other. However, when Ni(II) diethylene diamine diacetic acid, a polar (water-soluble) relaxation agent, is added to the membrane suspension, due to its collisions with the nitroxide moiety of CSL at the lipid/water interface (Fig. 6b), the SR EPR signal has two components when the Chol content in POPC bilayer exceeds 50 mol%. This demonstrates that in different cases, different relaxation agents should be used.

**Figure 7.**
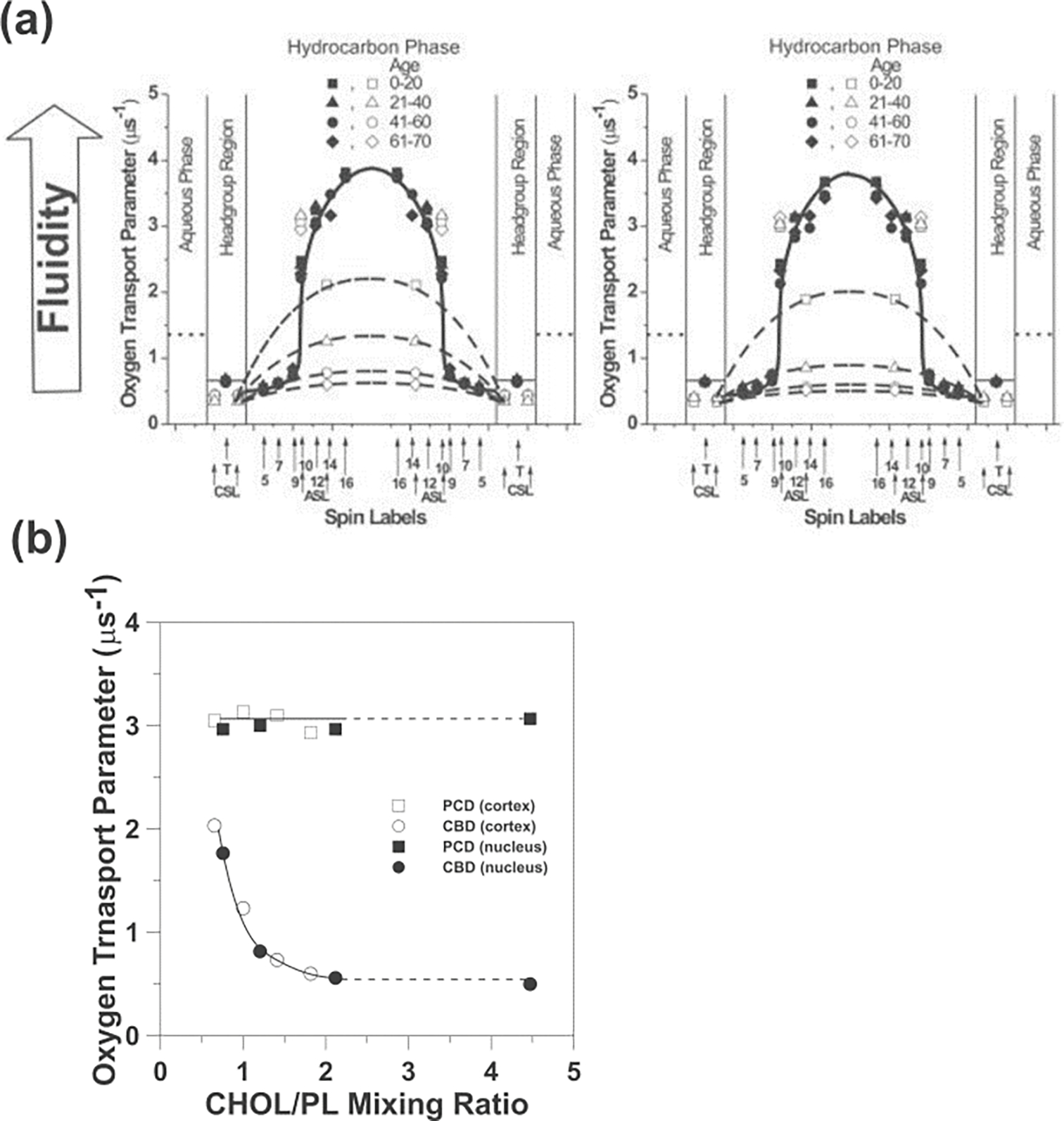
**(a)** Transmembrane profiles of the OTP for LLMs obtained from human donors of different age groups. Profiles were obtained at 37°C for LLMs prepared using the rapid solvent exchange method [85]. Profiles obtained with the PL-analog spin labels (filled symbols) are not contaminated by the presence of CBDs. Data obtained with Chol-analog spin labels (open symbols) are also included, showing that the CBDs are present in LLMs from all age groups. Thus, the PL bilayers in LLMs are always saturated with Chol. Approximate locations of the nitroxide moieties of spin labels are indicated by arrows. The OTP value in water is shown by dotted lines. **(b)** The OTP obtained with ASL in cortical and nuclear LLM plotted as a function of Chol content in these membranes (expressed as the Chol/PL mixing ratio). At a Chol/PL mixing ratio of ~2, the Chol crystals are formed. Thus, the Chol/PL molar ratio in phospholipid bilayers and the amount of Chol forming CBDs should not increase further. Chol crystals were detected in nuclear LLMs from the age group comprising 61–70 years (with a Chol/PL ratio of 4.4). Fig. 7.a is reproduced from Ref. [85]. Copyright 2022, with permission from Taylor and Francis. Data for Fig. 7.b are adapted from Ref. [85] [93, 94].

The CBD, which forms in the *l*_o_ phase bilayer cannot be treated as a separate phase—the *l*_o_ phase with the CBD is a structured or a dispersed *l*_o_ phase (see [56,59] for more explanations). Because (1-palmitoyl-2-(n-doxylstearoyl)phosphatidylcholines (n-PCs) and n-doxylstearic acid spin labels (n-SASLs) do not partition into CBDs (Fig. 4b), they report on only the properties of the bulk Chol saturated POPC (1:1 POPC:Chol molar ratio) bilayer that surrounds the CBDs. As was indicated in Sect. 7.1, the *l*_o_ phase has very different properties at the saturating Chol content. This feature helps in the study of various properties of model membranes made of the total lipids extracted from the plasma membranes of fiber cells of the eye lens (called lens lipid membranes [LLM]) [85]. These membranes are overloaded with Chol and, in the case of the human lens, the Chol content always exceeds the Chol saturation limit, ensuring that CBDs are always present in these membranes [85] and their surrounding environment is saturated with Chol. As was shown for single PL [29, 86–88] and mixed-PL bilayers saturated with Chol [85], their OTP profiles are practically identical independent of the PL composition of the bilayers. This is also evident from the OTP profiles for LLM from human lenses of donors of different age groups [85]. The PL composition of these membranes changes drastically with age; the glycerolipid content decreases and the sphingolipid content increases to exceed two-thirds of the total PLs for older donors [89–92]. Independent of these drastic changes, the OTP profiles across these membranes are identical (see Fig. 7a). Likewise, this applies to membranes without the integral proteins and possibly also to bulk lipid domains in biological membranes (see Sect. 8).

As shown in Fig. 7a, the Chol-analog spin label ASL can discriminate CBDs, giving two values for the OTP. Even though in LLMs obtained from donors of different ages, the profiles of the OTP across the membrane surrounding the CBDs are the same, and the profiles across CBDs (obtained with ASL and CSL) are different. This was surprising because the CBDs are pure Chol domains and their physical properties should be constant. This puzzle can be explained with help of a plot showing the OTP values as a function of Chol content in LLMs (Fig. 7b). As stated above, the OTP values obtained for ASL located in the membrane outside the CBDs do not change with an increasing Chol content. This indicates that this membrane region remains saturated with Chol even though the Chol content increases. When the Chol content in the membrane slightly exceeds 50 mol%, the CBDs that form there are small domains that are strongly affected by the surrounding bulk membrane. This happens because Chol exchanges between CBDs and the bulk membrane. The OTP values for CBDs and the surrounding lipids are then close to each other. At higher Chol contents, individual CBDs increase in size, and the effect of their surrounding decreases. The conclusion of this CBD study is that the amount of Chol that forms CBDs is equal to the amount of the excess Chol needed to saturate the lipids surrounding them, and that CBDs can be different sizes (see [85] for more explanation).

### 7.3. Boundary and Trapped Lipid Domains Induced by Membrane Integral Proteins

To investigate the organization and the dynamics of the lipids in membranes containing integral membrane proteins, a methodology employing O_2_ as a probe molecule—called the DOT method—was developed and is described in Ref. [28]. The investigated system was a DMPC bilayer with varying concentrations of BR. In reconstituted membranes of BR and DMPC containing trimers and oligomers of trimers of BR, the values of the recorded transmembrane profiles of the OTP were very low. This indicates that a new kind of lipid domains were formed there. The domains were called SLOT domains. It was concluded that a SLOT domain most likely consisted of lipids that were in contact with two integral proteins simultaneously or in contact with an integral membrane protein and the boundary lipids. In biological membranes, the SLOT domains are called trapped lipid domains. The boundary lipids, which exist in reconstituted membranes containing monomers of BR [28], cannot be discriminated from the bulk lipids using SR EPR technique because the exchange rate of lipids between these two domains is faster than the $T_{1^{-1}}\left(>10^{7}\;{\text{s}}^{-1}\right)$ of lipid spin labels in these domains (see also [95, 96]). As a result of this fast exchange, the OTP value in these membranes is smaller (by about 1.6 times) than that in the pure DMPC membrane. The advantages and limitations of the DOT method are schematically illustrated in Fig. 4 and described in the Fig. 4 caption (see also Sect. 5 and Eqs. 5 and 6 therein).

In studies of model lipid bilayers (including LLMs) and reconstituted membranes, all lipid spin labels presented in Fig. 1 of Ref. [71] and in Fig. 2 can be used. However, in the investigation of biological membranes, only n-SASLs and ASL can be incorporated there without damaging the sample by solvents dissolving these lipid spin labels in model membrane studies. To avoid damaging the sample, n-SASLs and ASL are introduced into biological membrane suspensions directly from a dry film of spin labels that is formed on the bottom of a test tube [49, 97]. It should be noted that n-SASLs do not participate into CBDs, so OTP profiles obtained with n-SASLs are not affected by the presence of CBDs. In biological membranes, n-SASLs are located in bulk lipids, boundary lipids, and trapped lipids. It was shown, using different techniques, that Chol molecules, as well as ASL, are largely excluded from the boundary lipids surrounding integral membrane proteins [98–103]. Thus ASL, which mimic the distribution and behavior of Chol, locates in CBDs, bulk lipid domain, and trapped lipid domain in biological membranes.

**Figure 8.**
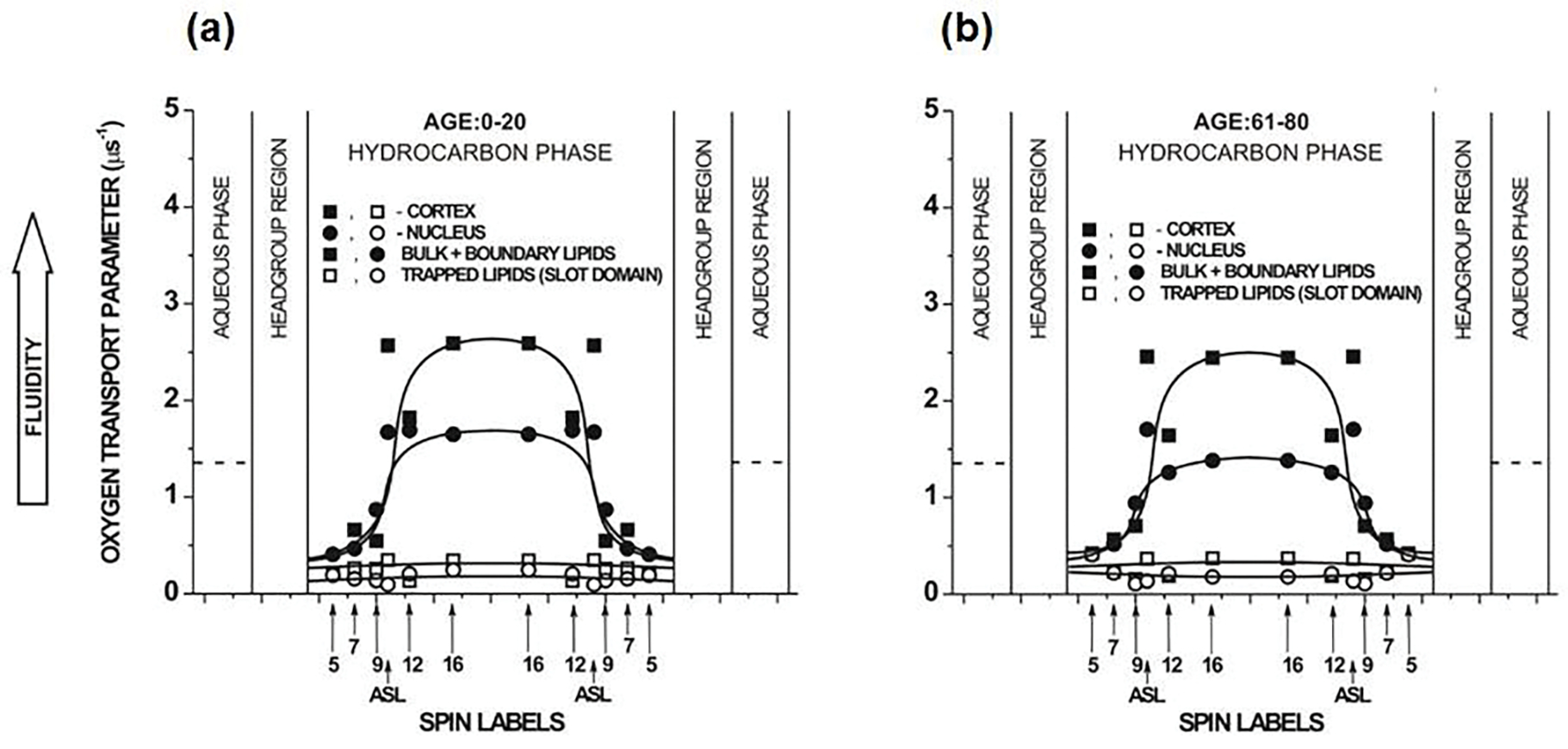
Profiles of the OTP across domains in intact cortical (■, □) and nuclear (●, ○) fiber cell plasma membranes of eye lenses from 0–20- and 61–80-year-old human donor groups. All profiles were obtained at 37°C. Profiles obtained with n-SASLs are not contaminated by the presence of CBDs. Profiles are reported for bulk plus boundary lipids (■, ●) and for trapped lipids (□, ○). Values obtained with ASL in domains of cortical and nuclear membranes are also included. Approximate localizations of the nitroxide moieties of spin labels are indicated by arrows. The OTP value in water is shown by dotted lines. Fig. 8 is reproduced from Ref. [70]. Copyright 2022, with permission from Elsevier.

The effects of integral membrane proteins on the organization of the lipid bilayer of intact biological membranes into domains and on the properties of these domains, investigated using O_2_ as a probe molecule and SR EPR as a technique, will be demonstrated for human fiber cell plasma membranes of eye lenses. The protein content in these membranes is extremely high, increases with the age of donor, and is different in lens cortical and nuclear membranes [104–109]. To clearly understand the impact of integral membrane proteins on the OTP profiles across intact membranes (such as those presented in Fig. 8), which can be obtained with n-SASs, the OTP profiles must first be obtained across the LLM (as those presented in Fig. 7a). It is assumed that any differences in the profiles are caused mainly by the integral membrane proteins.

The double exponential fits of SR EPR signals for all n-SASLs (*n* changes from 5 to 16) in cortical and nuclear membranes indicate that two environments with different OTP exist in these membranes (see Fig. 8 and the associated figure caption). The profiles with the greater OTP values were assigned to the bulk plus boundary lipid domains, whereas those with the smaller values were assigned to the trapped lipid domain, also called the SLOT domain.

The bulk domain, which is a lipid bilayer of the biological membrane not affected by the integral membrane proteins, is expected to have properties (including OTP profiles) close to those of a relevant LLM. However, the fast exchange of lipids between bulk and boundary lipids significantly decreases the OTP values recorded in cortical and nuclear membranes. The effect is stronger in nuclear than cortical membranes and increases with the age of donor (compare profiles in Fig. 8 with those presented in Fig. 7a). These most likely result from the higher protein content in lens nuclear membranes than in cortical membranes, and the increase in protein content with age. However, the bell shape of the profiles is preserved by a clear, abrupt increase in the OTP value at membrane locations deeper than the C9 atom of the acyl chains. The OTP profile across the trapped lipid domain (Fig. 8) indicates that phospholipids in this domain are tightly packed, with fewer vacant pockets, which facilitate the movement of O_2_. Also, the *trans-gauche* isomerization along the acyl chains in this domain is suppressed to that in the gel-phase membrane.

The double fits of SR EPR signals for ASL in cortical and nuclear intact membranes also indicate two environments of the spin label, each with a different OTP value. These OTP values are included in profiles presented in Fig. 8. Recalling the restriction for the ASL location in the membrane (see the second paragraph of this section), the greater values of OTP are assigned to the bulk domain plus CBD, while the smaller values are assigned to the trapped lipid domain. The OTP values measured with ASL in a more fluid environment (i.e., in the bulk domain plus CBD) are significantly smaller than those measured with ASL in the LLMs, which should have properties of the bulk domain anyway (compare Figs. 7a and 8). These values (measured with ASL in the bulk domain plus CBD) are significantly larger than those measured at the same membrane depth with n-SASL in the bulk plus boundary domains. The smaller OTP values measured with ASL coincide exactly with the profiles across trapped lipid domains (Fig. 8). As described in Sect. 7.2, ASL was successfully used to discriminate CBDs in simple model membranes and LLMs, although discrimination of CBDs in intact membranes was problematic. Fortunately, a method for detecting CBDs in intact biological membranes was developed recently and applied in studies of eye lens fiber cell plasma membranes [110]. This method is based on the DOT approach and uses ASL as a probe. The results obtained with the new method confirm the correctness of the previous assignment of components of the ASL SR EPR signal measured in the presence of O_2_.

## 8. Unique Information Obtained from Profiles of OTP Across Membrane Domains

As described in Sect. 6, O_2_ diffusion in the membrane occurs due to the presence and propagation of vacant pockets transiently created between lipid molecules in the bilayer. As was shown for membranes containing high amounts of Chol, OTP (O_2_ diffusion-concentration product) values increase abruptly at the membrane depth corresponding to the length of the C9–C10 bond in the acyl chain (1.3 Å) of PL. This precision may be treated as an atomic resolution of the method. Water, as another probe molecule, determines the profile of the membrane hydrophobicity [111]. This profile also shows an abrupt change at the membrane depth corresponding to the position of the C9–C10 bond in the membrane containing saturating amount of Chol [111]. However, the hydrophobicity profiles are obtained for frozen membranes where lipid motions are suppressed. This is in contrast with the OTP profiles that are obtained at physiological temperatures for fluid phase membranes. Because the abrupt increase in the OTP values occurs within a very narrow range of distances (Fig. 5c), the PLs and Chol in these fluid membranes saturated with Chol must be well aligned with one another, and their vertical fluctuation must be minimal. This conclusion was confirmed by molecular dynamic simulation of the Chol/POPC bilayer containing 50 mol% Chol [112].

Another unique feature of O_2_ as a probe molecule is that it allows OTP profiles to be obtained for coexisting domains without the need of their physical separation. Thus, each of the coexisting domains can be characterized by the physical parameter, i.e., the oxygen diffusion-concentration product. This parameter is not only significant for understanding oxygen diffusion within and across model and biological membranes, but also for understanding chemical reactions involving O_2_ that take place in different membrane environments (see for the review [113]). The rates of chemical reactions involving O_2_ depend on the local oxygen diffusion-concentration product, which can change drastically from one domain to another and also with membrane depth. Some reactions proceed more readily in membranes than in water. This is the case for reactions of O_2_ with nitric oxide, for which the acceleration factor is about 30 times [114, 115]. This is consistent with profiles of the diffusion concentration products for O_2_ [113] and nitric oxide [116] across lipid bilayers. Another class of chemical reactions that involves O_2_ and occurs in membranes includes lipid peroxidation and formation of reactive oxygen species [117, 118].

## 9. Other Applications of O_2_ as a Probe Molecule in EPR Spin-Labeling Studies of Membranes

It was shown [119] that the logarithm of the ratio of the collision rates of O_2_ and Ni(II) bis(acetyl acetonate) with a nitroxide moiety of the lipid-analog spin label is a linear function of the depth in the membrane at which the nitroxide moiety is located. This forms the basis of the collision gradient method for determining the depth in the membrane at which the spin labeled amino acids of integral membrane proteins are located. The requirement of this method is that the spin labeled amino acid must be located at the protein surface accessible to collisions with relaxation agents from the lipid phase. This method does not require the sample to be frozen, and measurements can be performed for fluid phase membranes at physiological temperatures. This collision gradient method, with O_2_ as a one of probe molecules, is routinely used in site-directed spin labeling [120].

The knowledge of the oxygen concentration and transport in tissues, cells, and subcellular structures like cellular membranes is central to understanding radiation [121–123] and photodynamic therapy [124, 125]. The EPR spin-label oximetry methods provide this information; in particular, these methods allow evaluation of the oxygen permeability coefficient across model and biological membranes. This evaluation is based on the profiles of OTP (and thus profiles of the oxygen diffusion-concentration product) across the investigated membranes (see Sect. 5); the details of the procedure are described in [126]. The evaluated values imply that lipid bilayers in the fluid phase are not barriers to oxygen transport. However, in biological membranes, especially those crowded with membrane proteins, lipids in the bilayers form different domains with different packing of lipid molecules (see Sect. 7.3). To evaluate the global oxygen permeability coefficient across such membranes, all components (i.e., oxygen permeability coefficients across all domains) must be evaluated. Because the oxygen permeability coefficient across trapped lipid domains and CBDs is much smaller than across the bulk fluid bilayer, plasma membranes crowded with integral membrane proteins and with high Chol content can form barriers to oxygen transport into cells. This knowledge is very significant for a new radiation therapy, named FLASH, that uses a very high doses of radiation[127–130]. Empirically, there seems to be a decrease in the side effects in normal tissue, while the therapeutic effect on tumors is not diminished. It is suggested that different barriers to the permeation of O_2_ across membranes in tumors and in normal tissue may be involved in the mechanism of FLASH. The physical interactions between O_2_ (the probe molecule) and nitroxide moieties of spin labels form basis of this method [131].

## 10. Final Remarks

All the above applications of the *T*_1_-sensitive EPR spin labeling oximetry methods were confined to the X-band EPR technique working at microwave frequency of 9.4 GHz. It was shown that for spin labels, *T*_1_ increases with the increase in the microwave frequency up to 35 GHz (Q-band spectrometer) [45]. However, its further increase up to 94 GHz (W-band spectrometer) [23, 132] causes a shortening of the *T*_1_. The longest spin label *T*_1_ measured at Q-band is favorable for all *T*_1_-sensitive methods, especially for spin label oximetry. The example of profiles of OTP obtained at Q- and W-band are presented and compared with those obtained at X-band in [23, 62, 133, 134]. Application of Q- and W-band techniques is advantageous over X-band for the study of small biological samples. The sample volume for the X-band SR EPR spectrometers equipped with the LGR is of 3 μL, while volumes for the Q- and W-band spectrometers are as small as 30 to 150 nL. Another significant advantage of Q- and W-band LGRs is a short spectrometer dead time, which is critical for discrimination of membrane domains (i.e., it should help to discriminate domains with high lipid exchange rates). The above information was added to notify readers about new capabilities of *T*_1_-sensitive EPR spin labeling oximetry methods at Q-band because not only X-band but also Q-band EPR spectrometers with SR capability are now commercially available from Bruker.
